# Veterinary Medical Society, University of Pennsylvania

**Published:** 1900-03

**Authors:** Harry E. Bender

**Affiliations:** Secretary


### VETERINARY MEDICAL SOCIETY, UNIVERSITY OF PENNSYLVANIA.

The meeting was called to order at eight o'clock p.m., January 12, 1900. Mr. Horner was appointed critic. It was moved, seconded, and carried to have 200 copies of the by-laws printed. A vote of thanks was extended to Dr. Pearson and Mr. Gillingham for the books which they contributed to the library; also a vote of thanks to the Banquet Committee for the faithful performance of their duties. Dr. Harger then gave a very interesting address on the subject of "Saline Transfusion." A vote of thanks was extended to Dr. Harger for his excellent address. The critic had no report to make. Adjourned at 9.30 p.m.

January 26th. The meeting was called to order at eight o'clock p.m. Mr. Powell was appointed critic. Next in order was the election of new officers. The result of the election was as follows: President, Mr. Cornman; Vice-President, Mr. Powell; Treasurer, Mr. Young; Executive Committee, Senior Class, Mr. Nolan; Junior Class, Mr. Walters, Mr. Watson; Freshmen Class, Mr. Paget; Librarian, Mr. Bassler.

We were next favored by an address by Dr. C. J. Marshall. A vote of thanks was extended to Dr. Marshall. Next in order was the Treasurer's report, which was accepted. The critic's report was very favorable. Adjourned at 9.30 p.m.

The meeting was called to order at eight o'clock p.m., February 9, 1900. Mr. Tallman was appointed critic. Mr. Andress was proposed and elected to membership.

Messrs. Young, Bassler, and Glass were appointed a committee to interview our several professors concerning subscriptions for the purpose of purchasing books for the library. Mr. Horner then favored the meeting by a paper on "Swine Plague and Hog Cholera." Next in order was a debate:

*Resolved*, That no stallion should be used for public service excepting such as has been examined and approved by a board of experts, the board to include a veterinarian and the examination to cover conformation and soundness.

Affirmative—Messrs. Young, Colton, and Glass. Negative—Messrs. Nolan, Gilliland, and McClintock. The judges, Messrs. Powell, Shore, and Zollinger, decided in favor of the negative side.

General debate followed and was very spirited, many taking part, as did also Drs. Pearson and Flower. The decision of the house was in favor of the negative side. A vote of thanks was extended to Drs. Pearson and Flower for the part they took in the general debate. Next was the Treasurer's report, which was accepted. The chair then appointed Mr. Shore and Mr. Glass on the Room Committee to fill the vacancies caused by the election to office of Messrs. Bassler and Paget. Mr. Hughes still serves as the member from the Senior Class.

It was moved, seconded, and carried to have the several pictures for the Reading-room framed.

A vote of thanks was extended to the following gentlemen for the books which they contributed to the library: Drs. Jacob, Hoskins, Huidekoper, and Mr. Mayer.

The critic's report was very favorable. Adjourned at 10 P.M.

The meeting was called to order at eight o'clock P.M., February 23, 1900. It was moved, seconded, and carried to dispense with the regular order of business. The meeting was then favored by an excellent address on the "Hackney Horse," by Mr. E. W. Twaddell. A vote of thanks was extended to Mr. Twaddell for his interesting and instructive address. Dr. Blount also gave a brief outline of his experience on board a transport from New Orleans to South Africa.

Adjourned at 10 o'clock P.M.

HARRY E. BENDER,
Secretary.

